# Supplementary material for: MetaRibo-Seq measures translation in microbiomes
Source: Nat Commun. 2020 Jun 29;11:3268. doi: 10.1038/s41467-020-17081-z (PMC7324362; doi:10.1038/s41467-020-17081-z)
Supplement: Supplementary file 10 — Supplementary Data 7 [file 41467_2020_17081_MOESM10_ESM.zip › File2/Confidence_High_Taxonomy/247309_out.krona.html]

Javascript must be enabled to view this page.

members
magnitude
magnitudeUnassigned
count
unassigned
taxon
rank

247309\_out

2

2
2
superkingdom

1239
phylum
2

186801
class
2

1
186802
2

SRS104693\_contig\_number\_contig-100\_4315.4316
order

541000
family
1

1263
genus
1


SRS019496\_contig\_number\_17784
1
species
40518
